# Effect of Alga *Gelidium* sp. Flour Extract on Lipid Damage Evolution in Heated Fish Muscle System

**DOI:** 10.3390/antiox11050807

**Published:** 2022-04-21

**Authors:** Roberta G. Barbosa, Marcos Trigo, Bin Zhang, Santiago P. Aubourg

**Affiliations:** 1 Department of Food Science and Technology, Federal University of Santa Catarina (UFSC), Rodovia Ademar Gonzaga, Florianópolis 88034-001, Brazil; rogarciafarma@hotmail.com; 2 Department of Food Technology, Marine Research Institute (CSIC), 36208 Vigo, Spain; mtrigo@iim.csic.es; 3 Key Laboratory of Health Risk Factors for Seafood of Zhejiang Province, College of Food Science and Pharmacy, Zhejiang Ocean University, Zhoushan 316022, China; zhangbin@zjou.edu.cn

**Keywords:** *Gelidium* sp., flour, aqueous extract, fish muscle, heating, lipids, primary oxidation, secondary oxidation, fluorescent compounds, hydrolysis

## Abstract

This research aimed to evaluate the antioxidant properties of flour obtained from red alga *Gelidium* sp., which is underutilised nowadays in food applications. Thus, a model system consisting of minced mackerel (*Scomber scombrus*) muscle and aqueous flour extract was subjected to heating treatment (50 °C) for 11 days. Resulting levels on conjugated diene (CD) and triene (CT) contents, peroxide value, thiobarbituric acid index, and fluorescent compound and free fatty acid (FFA) formation were monitored at different heating times. As a result, the presence of the aqueous extract of the alga flour led to higher levels (*p* < 0.05) of primary lipid oxidation compounds (CD and CT assessment) and lipid hydrolysis (FFA content). Contrarily, alga flour addition led to lower (*p* < 0.05) fluorescent compound formation measured in the aqueous and organic fractions resulting from the lipid extraction of the fish muscle and in the supernatant medium corresponding to the heating reaction system. All effects were found to be more important (*p* < 0.05) with increased alga flour concentration and heating reacting time. According to the straight relationship between the interaction compound formation and nutritional and sensory values, this study opens the way to the quality enhancement of thermally treated seafood by the addition of flour extract from *Gelidium* sp.

## 1. Introduction

The thermal treatment of marine species constitutes an important and traditional means of seafood preservation that leads to a wide range of nutritional and delicious food [1,2]. However, according to the thermal sensitivity of a wide number of chemical constituents and nutrients, different detrimental effects resulting from heat treatment have been mentioned such as the oxidation of lipids and vitamins, the degradation of nutrients, and the leaching of water-soluble proteins, vitamins, and minerals [3,4]. Consequently, an important detrimental effect on nutritive and sensory qualities can be produced if thermal processing conditions are not previously optimised or do not include the addition of accurate preservative substances [5,6].

The lipid fraction of marine species is now the subject of much attention as a result of its high content on ω-3 polyunsaturated fatty acids (PUFA), which have shown a positive effect in preventing several human diseases [7,8]. However, this lipid fraction has been shown to be highly susceptible to quality loss through lipid oxidation. The onset of rancidity development can be fast, particularly in fatty species, in whose muscle large amounts of pro-oxidant molecules (i.e., metalloproteins and endogenous enzymes) and lipids coexist [9,10]. The use of antioxidants has shown to be an effective way to minimise or prevent lipid oxidation development in seafood, thus retarding the formation of toxic oxidation products, maintaining nutritional quality, and enhancing the shelf-life of seafood [11]. On the basis of safety concerns, great efforts are being made nowadays to replace synthetic antioxidants with others obtained from natural resources [12].

For a long time, marine algae have been considered as part of the human diet in different Asian countries. Among macroalgae, red macroalgae have shown a remarkable level of constituents exhibiting high nutritional value [13], and constitute a rich source of preservative compounds (phenolic compounds, flavonoids, carbohydrates, and others), including antioxidant [14,15] and antimicrobial [16] properties. Most red macroalgae are primarily known for their industrial use for extracting phycocolloids (algin, furcellaran, agar, and carrageenan) [17,18], which are used to enhance the physical properties of edible films in novel food packing applications [19,20]. Remarkably, recent studies have proved an antioxidant effect when red alga extracts have been present during the heat treatment of seafood [21,22]. Interestingly, marine algae have been considered by the European Council Regulation [23] as a food or food ingredient, so their use would not pose a health problem for consumers.

The present research aimed to analyse, to our knowledge for the first time, the antioxidant properties of flour obtained from the red alga *Gelidium* sp., an organism commonly employed for animal feeding but poorly studied and underutilised in food applications. For it, a model system consisting of minced mackerel (*Scomber scombrus*) muscle and aqueous flour extract was subjected to heating treatment (50 °C) for 11 days. The resulting lipid oxidation (levels of conjugated dienes and trienes, peroxides, thiobarbituric acid reactive substances, and fluorescent compounds) and hydrolysis (free fatty acid value) development were monitored at different heating times.

## 2. Materials and Methods

### 2.1. Raw Fish, Starting Alga Flour, and Preparation of Flour Extract

Fresh Atlantic mackerel (*S. scombrus*) (12 individuals) (weight range: 235–280 g; length range: 28–34 cm) were acquired at Vigo harbour (North-Western Spain) and transported on ice to the laboratory. Then, fish were randomly divided into three (*n* = 3) groups (4 individuals per group), eviscerated, beheaded, and filleted. In each group, the white muscle of fish fillets was minced and homogenised.

Commercial flour obtained from *Gelidium* sp. was provided by Industrias Roko S. A. (Llanera, Asturias, Spain). The flour exhibited the following proximate composition (%): 12.2 (moisture), 31.5 (protein), 0.2 (lipids), 14.3 (ash), and 42.8 (total carbohydrate). Additionally, the fatty acid (FA) profile was analysed according to previous research [24]. Individual FA composition was as follows: 5.0 ± 0.3 (C14:0), 52.3 ± 3.4 (C16:0), 2.4 ± 0.2 (C16:1ω7), 1.7 ± 0.1 (C18:0), 8.1 ± 0.5 (C18:1ω9), 1.5 ± 0.1 (C18:1ω7), 1.3 ± 0.1 (C18:2ω6), 0.4 ± 0.1 (C20:1ω9), 16.0 ± 1.2 (C20:4ω6), 11.0 ± 0.9 (C20:5ω3), 0.1 ± 0.0 (C22:5ω3), and 0.1 ± 0.0 (C22:6ω3).

A mixture of alga flour (26 g) and distilled water (400 mL) was stirred for 30 s, sonicated for 30 s, and centrifuged at 3500× *g* for 30 min at 4 °C. The supernatant was then recovered, and the extraction process was repeated three more times. Finally, all four supernatants were pooled together and made up to 2 L with distilled water.

### 2.2. Flour Extract/Mackerel Muscle Model System

For the preparation of the model system, 4 g portions of minced mackerel muscle were placed in 30 mL glass tubes and mixed with 0.5, 1.5, and 4.5 mL of the above-mentioned alga flour extract, respectively. Then, 9.5, 8.5, and 5.5 mL of distilled water were added, respectively, leading to RAE-1 (low-concentrated), RAE-2 (medium-concentrated), and RAE-3 (high-concentrated) batches. The control (CTR) batch was prepared by mixing 4 g of mackerel muscle and 10 mL of distilled water.

Mixtures corresponding to all batches were firmly closed and stirred for 30 s, sonicated for 30 s, and incubated at 50 °C for 11 days. Sampling was undertaken at 0, 1, 4, 7, and 11 days. At each sampling time, samples were cooled to room temperature (18–20 °C) and centrifuged. Then, the supernatant was collected for fluorescence analysis and the solid phase (i.e., fish muscle) was subjected to lipid extraction for lipid damage assessment. Three independent sets were carried out that were analysed independently (*n* = 3).

With the aim of showing the antioxidant properties of alga flour extract, several previous trials were carried out. In such trials, different heating temperatures and times and different fish muscle/alga extract ratios were checked in order to carry out the study under such experimental conditions where a relevant lipid oxidation development was produced and the antioxidant properties of the alga extract could be proved.

### 2.3. Lipid Damage Detection

Lipid extraction was carried out on the mackerel white muscle by using the Bligh and Dyer [25] method; this procedure employs a chloroform/methanol (1/1, *v*/*v*) mixture. Lipid quantification was carried out according to the Herbes and Allen [26] method. Lipid content was calculated as g·kg^−1^ mackerel muscle.

Conjugated diene (CD) formation was measured spectrophotometrically (Beckman Coulter DU 640, Spectrophotometer, Beckman Coulter Inc., Brea, CA, USA) at 233 nm [27] on the lipid extract. Results are expressed by applying the following formula: CD = A × V·w^−1^, where A is the absorbance reading, and V and w denote the volume (mL) and the weight (mg) of the corresponding lipid extract, respectively.

Conjugated triene (CT) formation was measured at 268 nm [27] on the lipid extract. Results are expressed by applying the following formula: CT = A × V·w^−1^, where A is the absorbance reading, and V and w denote the volume (mL) and the weight (mg) of the corresponding lipid extract, respectively.

The peroxide value (PV) was determined spectrophotometrically (520 nm) on the lipid extract. For it, peroxide reduction with ferric thiocyanate was carried out [28]. Results were calculated as meq. active oxygen·kg^−1^ lipids.

The thiobarbituric acid index (TBA-i) was assessed by employing the Vyncke [29] procedure. For it, the formation of thiobarbituric acid reactive substances (TBARS) was spectrophotometrically assessed at 532 nm and calculated from a standard curve employing 1,1,3,3-tetraethoxy-propane (TEP). Results were calculated as mg malondialdehyde·kg^−1^ muscle.

The free fatty acid (FFA) value was assessed on the lipid extract of the fish muscle by the Lowry and Tinsley [30] procedure. This method is based on complex formation with cupric acetate-pyridine followed by spectrophotometric (715 nm) assessment. Results were calculated as g FFA·kg^−1^ muscle.

### 2.4. Interaction Compound Detection

Interaction compounds produced by the reaction of oxidised lipids and protein-type molecules were determined by fluorescence spectroscopy (Fluorimeter LS 45; Perkin Elmer España; Tres Cantos, Madrid, Spain). The formation of fluorescent compounds was measured at 393/463 and 327/415 nm, according to previous research [31]. For it, the relative fluorescence (RF) was determined as follows: RF = F/F_st_, where F is the fluorescence measured at each excitation/emission wavelength pair and F_st_ is the fluorescence intensity of a quinine sulphate solution (1 µg·mL^−1^ in 0.05 M H_2_SO_4_) at the corresponding wavelength pair. Fluorescent compound formation was determined as the fluorescence ratio (FR) between the two RF values: FR = RF_393/463 nm_/RF_327/415 nm_.

In the present study, the fluorescent compound formation was analysed on different substrates of the flour extract/minced muscle model system. First, it was studied on the aqueous (FR_aqu_) and organic (FR_org_) phases obtained from the muscle lipid extraction [25]. Then, it was determined in the supernatant aqueous medium (FR_med_) resulting from the heating reaction of fish muscle in the presence of alga flour extract.

### 2.5. Statistical Analysis

Once values obtained for the different chemical parameters verified the normal distribution (Kolmogorov–Smirnov test), parametric analysis was carried out. The one-way ANOVA method was used to explore differences resulting from the effect of flour extract concentration and heating time. For this, the least-squares difference (LSD) method was employed to perform the comparison of means. As expressed above, three replicates (*n* = 3) were developed in the present research. Analyses were carried out by applying the PASW Statistics 18 software for Windows (SPSS Inc., Chicago, IL, USA), and a confidence interval at the 95% level (*p* < 0.05) was considered.

Correlation analyses between lipid damage indices and heating time were analyzed by using the Pearson test.

## 3. Results

### 3.1. Evolution of Primary and Secondary Lipid Oxidation

An increasing trend of CD value with heating time was proved in all kinds of samples (*r* = 0.93–0.95), with all values being included in the 1.0–2.3 range (Table 1). At the 4–11-day heating time, the highest average CD values were detected by increasing the presence of the flour extract in the reacting medium. Remarkably, significant differences (*p* < 0.05) with control were observed on days 7 and 11 in RAE-2 and RAE-3 batches. Consequently, the flour extract presence has led to an increased level of CD value in fish muscle, with this effect being more remarkable when increasing the extract concentration and the reacting time.

As for CD assessment, CT content indicated a continuous increase with heating time in all kinds of samples (*r* = 0.91–0.95) (Table 1). For this kind of lipid oxidation compound, the highest average values were obtained in all cases at the end of the study. A comparison among batches showed increasing average values when increasing the flour extract presence in the heating system. Compared to control samples, significantly higher (*p* < 0.05) values were detected in the RAE-3 batch (days 1, 7, and 11) and in the RAE-2 batch (days 7 and 11). Therefore, a remarkable retention of this kind of lipid oxidation compound was proved by flour extract presence in the reacting medium, with this effect increasing with the extract concentration and the heating time.

Peroxide formation in the present study can be considered low; thus, all values remained below a score of 1.90 (Table 2). For all batches, a definite trend of PV evolution with heating time could not be concluded. Remarkably, the highest average values were obtained on day 7 in all cases. Significant differences (*p* > 0.05) were not observed between control and flour-treated samples. Therefore, no effect of the flour extract presence on this kind of lipid oxidation compound in fish muscle could be demonstrated.

A general formation (*p* < 0.05) of TBARS was detected in all batches during the 0–4-day period. After this time, a decrease of the average TBA-i was detected in all kinds of samples, except for the batch including the highest flour extract concentration. For this batch, an increasing trend in TBARS content was maintained till the end of the study. A comparison among batches revealed scarce differences; thus, the highest average values were obtained in RAE-2 batch (day 1), CTR batch (day 4), and RAE-3 batch (7–11-day period). Compared to the control, lower (*p* < 0.05) values were obtained on day 4 in the RAE-1 batch and higher (*p* < 0.05) on day 11 in the RAE-3 batch. It is concluded that the presence of the flour extract in the reacting medium did not lead to a definite trend (*p* > 0.05) on the level of this kind of secondary lipid oxidation compounds in fish muscle.

### 3.2. Fluorescent Compound Formation

**Figure 1:**
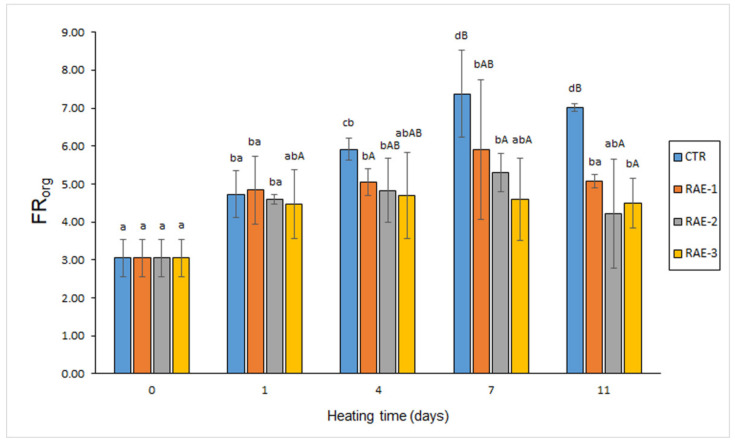
The evolution of fluorescent compound content in the lipid extract of fish muscle (FR_org_) heated in the presence of red alga extract (RAE). Abbreviations of RAE concentrations are as expressed in Table 1. Mean values of three independent determinations (*n* = 3); standard deviations are shown by bars. Mean values accompanied by lowercase (a–d) letters denote significant differences (*p* < 0.05) as a result of heating time; mean values accompanied by capital letters (A,B) denote significant differences (*p* < 0.05) as a result of RAE concentration.

For CTR, RAE-1, and RAE-2 batches, a progressive fluorescent compound formation (*p* < 0.05) in the lipid extract of the fish muscle (FR_org_) was detected for the 0–7-day period, which was followed by a decrease of the average value at the end of the experiment (Figure 1). For samples corresponding to the RAE-3 batch, an average value increase was detected for the 0–4-day period, followed by a decrease till day 11. A comparison of the samples revealed an inhibitory effect (*p* < 0.05) of flour extract presence on the formation of these deteriorative compounds in the 4–11-day heating time. During this period, the highest average values were detected in the control batch; differences with treated-muscle batches were found to be significant (*p* < 0.05) on day 4 (RAE-1 batch), day 7 (RAE-2 and RAE-3 batches), and day 11 (all flour-containing batches).

**Figure 2:**
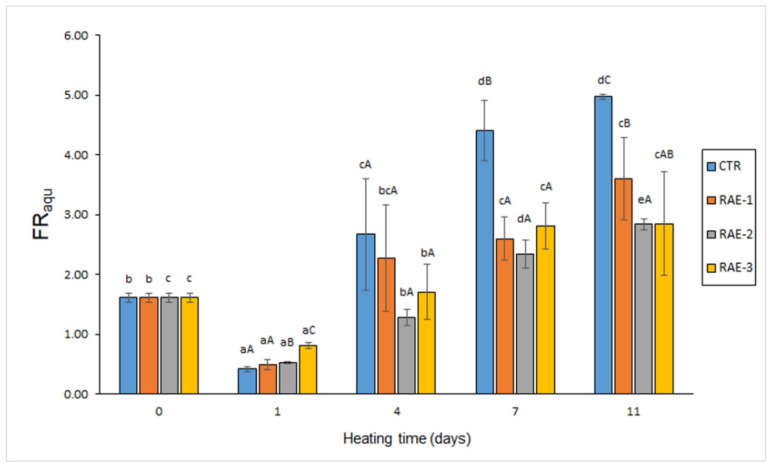
The evolution of fluorescent compound content in the aqueous extract obtained from the lipid extraction of fish muscle (FR_aqu_) heated in the presence of red alga extract (RAE). Abbreviations of RAE concentrations are as expressed in Table 1. Mean values of three independent determinations (*n* = 3); standard deviations are shown by bars. Mean values accompanied by lowercase (a–e) letters denote significant differences (*p* < 0.05) as a result of heating time; mean values accompanied by capital letters (A–C) denote significant differences (*p* < 0.05) as a result of RAE concentration.

The fluorescent compound content measured in the aqueous fraction (FR_aqu_) resulting from the lipid extraction of muscle indicated a marked decrease (*p* < 0.05) in all kinds of samples on day 1 (Figure 2). Contrarily, a progressive increase of this quality parameter was observed for the 4–11-day period in all batches. A good correlation value was detected between heating time and content of this kind of fluorescent compound (*r* = 0.91–0.94). A comparison among batches revealed two different trends. Thus, on day 1, an increased FR_aqu_ was observed by increasing the flour extract presence in the heating medium. Contrarily, at advanced heating times (i.e., 4–11-day period), lower average values were detected in RAE-2 and RAE-3 batches than in CTR and RAE-1 batches. For this reacting period, differences in the control batch were significant (*p* < 0.05) on days 7 and 11 for all flour-containing batches. Remarkably, the lowest average values were detected in samples corresponding to the RAE-2 batch. Therefore, an inhibitory effect of flour extract presence was implied on the formation of fluorescent compounds measured in the aqueous phase resulting from the lipid extraction of the mackerel muscle.

**Figure 3:**
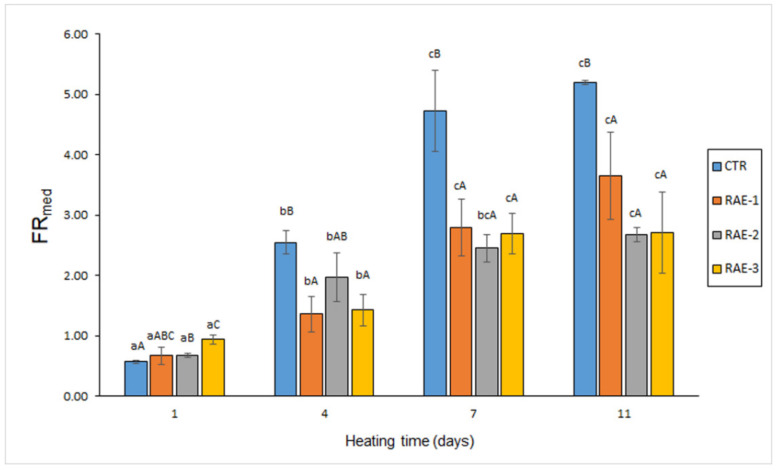
The evolution of fluorescent compound content in the liquid medium (FR_med_) resulting from fish muscle heating in the presence of red alga extract (RAE). Abbreviations of RAE concentrations as expressed in Table 1. Mean values of three independent determinations (*n* = 3); standard deviations are shown by bars. Mean values accompanied by lowercase (a–c) letters denote significant differences (*p* < 0.05) as a result of heating time; mean values accompanied by capital letters (A–C) denote significant differences (*p* < 0.05) as a result of RAE concentration.

A progressive formation (*p* < 0.05) of fluorescent compounds with heating time (*r* = 0.89–0.94) was detected in the aqueous medium (FR_med_) of the reacting system in all batches (Figure 3). As for FR_aqu_, a general increase of the content of this kind of fluorescent compounds was observed throughout the 4–11-day period. Remarkably, the flour extract presence provoked an inhibitory effect (*p* < 0.05) on the formation of this kind of compounds in the 4–11-day period. Thus, a comparison with the control led to lower (*p* < 0.05) values on day 4 (FE-1 and FE-3 batches) and on days 7 and 11 (all flour-containing samples).

### 3.3. Evolution of Lipid Hydrolysis

In all kinds of samples, a remarkable FFA content increase was detected at advanced times of heating reaction (*r* = 0.91–0.94) (Table 3). Thus, a remarkable formation of FFA was detected on day 7 in fish samples corresponding to CTR, RAE-1, and RAE-2 batches; in the case of the RAE-3 batch, a substantial increase was observed on day 4. For the 4–11-day period, the average values found in the RAE -3 batch were higher than their counterparts from other batches; notably, the differences between this batch and the CTR one were significant (*p* < 0.05) for this period. Furthermore, the samples corresponding to the RAE-1 and RAE-2 batches also showed higher (*p* < 0.05) FFA levels than the control fish for the 7–11-day period. Therefore, a significant retention (*p* < 0.05) of FFA content in fish muscle was concluded by the presence of the flour extract in the heating medium, with its effect increasing with the extract concentration and the heating time. 

## 4. Discussion

### 4.1. Lipid Oxidation Development in the Present Study

The present results have shown a strong effect of alga flour extract on the lipid oxidation development of mackerel muscle. Thus, primary lipid oxidation compounds (i.e., CD and CT) showed higher levels (*p* < 0.05) in the treated-fish muscle when compared with the control. Contrarily, lower levels (*p* < 0.05) of interaction compound formation between oxidised lipids and protein-like compounds were detected in the treated-fish samples in comparison with the CTR batch. Remarkably, such effects were shown to be more important (*p* < 0.05) when increasing the flour extract presence in the reacting medium and increasing the heating time. 

The lipid oxidation of food in general can be considered a complex deteriorative mechanism including the formation of different kinds of compounds, with some of them being unstable and susceptible to breakdown and forming lower-weight compounds and/or reacting with other molecules, and most of them being of the nucleophilic type, which is present in fish muscle [32,33]. When a heat treatment is applied as in the present case, lipid oxidation evolution would depend on several factors [3,4]. One the one hand, heat treatment would give rise to an increase of the formation of such compounds as a result of the oxidation of unsaturated lipids; in the case of marine species, this factor should be highly important on the basis of the highly unsaturated lipid composition. On the other hand, the heat treatment itself would facilitate and accelerate the breakdown of oxidation compounds into low-molecular-weight molecules (i.e., aldehydes, ketones, etc.), which are more reactive than primary compounds. Finally, following the formation and breakdown of primary and secondary lipid oxidation compounds, the interaction of the resulting molecules with nucleophilic substrates (i.e., protein-type compounds) present in the current fish muscle system would facilitate fluorescent compounds formation.

Previous research has reported low values of primary and secondary lipid oxidation compounds content during thermal treatment of seafood. Thus, similar peroxide and TBARS levels to those in the current studies have been obtained in canned Atlantic Chub mackerel (*Scomber colias*) [24], canned Atlantic mackerel (*S. scombrus*) [34], and canned salmon (*Salmo salar*) [21]. Remarkably, the assessment of fluorescent compound formation during the thermal processing of seafood (i.e., cooking and canning) was shown to be a reliable analytical tool to assess quality loss during seafood processing [31].

According to previous research [35,36], such interaction compounds can have a different hydrophilic/lipophilic character. Thus, interaction compounds presenting a relevant lipophilic framework would remain in the organic phase during lipid extraction, while fluorescent substrates formed from oxidised membrane lipids with amino-type compounds would remain attached to the amino constituent and therefore would be present in the aqueous phase during lipid extraction. Therefore, as in the current study (Figure 1 and Figure 2), measurement of both kinds of extracts would be necessary to ensure a complete assessment of the interaction compound formation. Furthermore, and according to the extraction capacity of water, hydrophilic fluorescent compounds could be partly present in the reacting medium of the present study, with this extraction process from the fish muscle being facilitated by the heating treatment. Consequently, the analysis of this liquid fraction in the present muscle system (Figure 3) has provided a useful tool to assess the interaction compound formation.

Previous research has shown that the formation of interaction compounds can have a remarkable effect on the nutritional and sensory values of seafood [31,37]. Thus, the increased formation of such compounds has led to important losses of essential amino acids (lysine, methionine, etc.), digestibility decrease, and a decrease of sensory acceptability (texture modification and the development of off-odour, off-flavour, and browning). On the basis of the current results, the addition of alga flour extract from *Gelidium* sp. may lead to nutritional and sensory quality retention during seafood thermal processing.

### 4.2. Antioxidant Activity of Red Alga Extracts

The antioxidant properties of red alga constituents have already been described, both in in vitro studies as well as in processed seafood. In such studies, different kinds of molecules have been found to be responsible for the preservative effect, according to the kind of extracting medium employed (i.e., water, ethanol, methanol, ethyl acetate, etc.). On the basis that a water extract of red alga flour was employed in the present study, the antioxidant activity found can be explained on the basis of the presence of the hydrophilic constituents present in the red alga flour.

Thus, and according to present results, previous in vitro studies have shown antioxidant properties of water extracts obtained from red macroalgae. Research has concerned red algae species such as *Hypnea flagelliformis* [38], *Gracilaria verrucosa* [39], and *Gracilaria gracilis* [40]. In all cases, the preservative behaviour was explained on the basis of the presence of phenolic compounds, flavonoids, carbohydrates, and alkaloids, which have the ability to neutralise free radicals. Among such preservative molecules, carbohydrates have deserved special attention. Remarkably, the antioxidant activity of polysaccharide compounds obtained from aqueous extracts of red algae has been detected in in vitro studies focused on *Porphyra yezoensis* [14], *Gracilaria corticata* [17], and *Spyridia hypnoides*, *Asparagopsis taxiformis*, *Portieria hornemannii*, and *Centroceras clavulatum* [41]. According to these studies, the current inhibition of lipid oxidation development could be explained on the basis of the remarkable presence of carbohydrate compounds that were shown to be present in the starting alga flour, according to the proximate composition analysis.

In agreement with the preservative role reported for carbohydrate compounds, previous research accounts for different attempts focused on the analysis of carbohydrate composition in different red algae. Thus, Zeid et al. [42] analysed the carbohydrate composition of hot- and cold-water extracts obtained from red alga *Pterocladia capillacea*; the HPLC analysis revealed enriched content on glucuronic acid, arabinose, and glucose (cold extract), whereas the hot extract was found to be rich in glucuronic acid and fructose. Later on, Olasehinde et al. [43] observed the presence of glucose, galactose, fucose, arabinose, and xylose as the main monosaccharides present in sulphated polysaccharides from *Gelidium pristoides*. Recently, Pei et al. [18] analysed the polysaccharide composition from *Gelidium amansii*; as a result, rhamnose, glucuronic acid, glucose, galactose, xylose, and L-fucose were found to be the main components.

Previous research accounts for the lipid oxidation inhibition and sensory acceptance increase of thermally treated seafood as a result of treatment with red alga extracts. Thus, Ortiz et al. [21] employed aqueous extracts of red algae *Pyropia columbina* and *Gracilaria chilensis* as filling media during salmon (*S. salar*) canning; the inhibition of lipid oxidation development (assessment of PV, *p*-anisidine value, and polyene index) and the retention of astaxanthin and tocopherol contents were observed in canned salmon resulting from the alga presence in the filling medium. Later on, cooked salmon (90 ± 5 °C for 30 min) was subjected previously to an aqueous extract dipping of red algae *Gigartina radula*, *Iridaea larga, Gigartina chamissoi, Gracilafia chilensis, Gelidium chilense,* and *Gigartina akottsbergii* [22]. As a result of this pre-treatment, lower lipid oxidation development was observed in fish muscle, as well as protection of end antioxidants (i.e., tocopherols and astaxanthin); this inhibitory effect was attributed to the presence of preservative molecules such as polyphenols, phlorotannins, diterpenes, carotenoids, and phytosterols in alga extracts.

### 4.3. Lipid Hydrolysis Development during the Present Study

A remarkable effect of alga flour extract on the FFA content was observed in the present study. As for the CD and CT compounds, a protective effect (*p* < 0.05) on FFA molecules was detected, with this being more relevant (*p* < 0.05) when increasing the flour extract concentration and at advanced heating times. 

The present FFA values can be considered the result of different effects. First, the heating treatment can lead to the hydrolysis development of high-molecular-weight lipid classes such as triacylglycerols (TG) and phospholipids (PL), as a result of heat catalysation [3,4]; consequently, this effect would lead to an increase of FFA content. On the other hand, FFA compounds are likely to be oxidised or broken down during heating treatment because they provide greater accessibility to pro-oxidant molecules such as oxygen when compared with TG and PL lipid classes [44]; therefore, this effect would lead to a FFA content decrease. Finally, the presence of antioxidant compounds included in the alga flour extract may protect FFA from oxidation and breakdown, so that the content decrease resulting from the above-mentioned second effect could be decreased. The fact that CTR samples show increasing values with heating times indicates that the first factor has been more important than the second one. However, the higher levels obtained in flour-treated fish than in the control indicated that the presence of the flour extract in the reacting medium has provided a preservative effect on lipid hydrolysis compounds.

Previous research concerning the effect of alga extracts on FFA content during seafood thermal processing can be considered scarce and is mostly focused on canned seafood. As in the present research, the inclusion of aqueous *B. bifurcata* extracts in the packing system employed for Atlantic mackerel (*S. scombrus*) canning led to a preservative effect of FFA in canned muscle [34]. Similarly, higher levels of FFA were detected in canned Atlantic Chub mackerel (*S. colias*) as a result of adding aqueous extracts of *F. spiralis* and *U. lactuca* in the filling medium employed [24].

## 5. Conclusions

The present results have shown a strong effect of *Gelidium* sp. extract on the lipid damage development of mackerel muscle. Thus, the presence of the aqueous extract of the alga flour led to higher levels (*p* < 0.05) of primary lipid oxidation (i.e., assessment of CD and CT) and FFA compounds. Contrarily, alga flour addition led to lower (*p* < 0.05) fluorescent compound formation, measured in the organic and aqueous fractions obtained from the muscle lipid extraction and in the supernatant medium corresponding to the heating reaction system. All effects were found to be more important (*p* < 0.05) when increasing the alga flour concentration and the heating reacting time. Notably, a definite trend on the peroxide and TBARS formation in fish muscle could not be concluded (*p* > 0.05). A protective effect on CD, CT, and FFA compounds is concluded, with this leading to the lower formation of interaction compounds between oxidised lipids and protein-like molecules. 

According to the straight relationship between interaction compound formation and the loss of essential amino acids, a digestibility decrease, and the development of off-odours and off-flavours, the enhancement of sensory and nutritional properties could be implied as a result of alga flour presence during seafood thermal processing. However, in order to apply the present alga flour extract to commercial thermally treated seafood, a previous optimisation of experimental conditions (i.e., seafood weight/alga extract ratio and particular processing conditions) is recommended to carry out.

It is concluded that the current study opens the way to a novel and beneficial employment of *Gelidium* sp. flour for the quality enhancement of thermally treated seafood. On the basis of the abundance of *Gelidium* sp., this procedure would agree with current global interests in food technology in the search for new strategies including preservative compounds obtained from natural sources and from sources underutilised in food applications. Further research is envisaged to optimise the extraction of preservative compounds and to analyse the molecules involved in this preservative action.

## Figures and Tables

**Table 1:** The formation of conjugated diene and triene compounds in fish muscle heated in the presence of red alga extract (RAE) *.

Quality Index	RAE Concentration	Heating Time (Days)				
		0	1	4	7	11
Conjugated dienes **	CTR	1.08 a (0.01)	1.20 bA (0.04)	1.29 cA (0.04)	1.39 cA (0.06)	1.49 dA (0.01)
	RAE-1	1.08 a (0.01)	1.28 bA (0.05)	1.31 bA (0.03)	1.49 cAB (0.08)	1.67 cA (0.26)
	RAE-2	1.08 a (0.01)	1.22 bA (0.02)	1.36 cA (0.06)	1.57 dB (0.06)	2.10 eB (0.06)
	RAE-3	1.08 a (0.01)	1.17 bA (0.03)	1.38 cA (0.10)	1.92 dC (0.25)	2.25 eB (0.15)
Conjugated trienes **	CTR	0.05 a (0.00)	0.07 abA (0.01)	0.10 bA (0.00)	0.10 bcA (0.01)	0.13 cA (0.01)
	RAE-1	0.05 a (0.00)	0.07 bA (0.00)	0.13 bcA (0.07)	0.20 cAB (0.09)	0.22 cA (0.11)
	RAE-2	0.05 a (0.00)	0.08 bAB (0.01)	0.22 cA (0.01)	0.30 dBC (0.04)	0.45 eB (0.02)
	RAE-3	0.05 a (0.00)	0.10 bB (0.01)	0.23 cA (0.02)	0.37 dC (0.09)	0.47 dB (0.09)

* Abbreviations: CTR (control batch); RAE-1, RAE-2, and RAE-3 correspond to batches including low, medium, and high concentrations of RAE, respectively. Mean values of three independent determinations (*n* = 3); standard deviations are shown in brackets. Mean values followed by lowercase letters (a–e) denote significant differences (*p* < 0.05) as a result of heating time; mean values followed by capital letters (A–C) denote significant differences (*p* < 0.05) as a result of RAE concentration. ** Units as expressed in the Material and Methods section.

**Table 2:** The assessment of peroxide value (PV) and the thiobarbituric acid index (TBA-i) in fish muscle heated in the presence of red alga extract (RAE) *.

Quality Index	RAE Concentration	Heating Time (Days)				
		0	1	4	7	11
PV (meq. active oxygen·kg^−1^ lipids)	CTR	0.39 a (0.18)	1.51 cA (0.11)	0.95 bA (0.18)	1.81 cA (0.60)	1.38 cA (0.10)
	RAE-1	0.39 a (0.18)	1.19 bA (0.30)	0.72 abA (0.14)	1.54 bA (0.37)	1.07 abA (0.53)
	RAE-2	0.39 a (0.18)	1.36 bcA (0.30)	0.81 abA (0.30)	1.68 cA (0.18)	1.14 bA (0.12)
	RAE-3	0.39 a (0.18)	1.32 cdA (0.21)	0.80 bA (0.04)	1.89 dA (0.34)	0.92 bcA (0.24)
TBA-i (mg malondi-aldehyde·kg^−1^ muscle)	CTR	1.29 a (0.67)	2.79 bcAB (0.66)	3.63 bcBC (0.70)	2.83 cA (0.12)	2.45 bA (0.21)
	RAE-1	1.29 a (0.67)	2.61 cA (0.19)	2.69 cA (0.17)	2.61 bcA (0.36)	2.07 bA (0.16)
	RAE-2	1.29 a (0.67)	3.02 bB (0.58)	3.55 bC (0.36)	2.85 bA (0.39)	2.50 abAB (0.99)
	RAE-3	1.29 a (0.67)	2.51 bA (0.38)	2.90 bcAB (0.19)	3.15 cA (0.56)	3.17 cB (0.18)

* Abbreviations of RAE concentrations as expressed in Table 1. Mean values of three independent determinations (*n* = 3); standard deviations are shown in brackets. Mean values followed by lowercase letters (a–d) denote significant differences (*p* < 0.05) as a result of heating time; mean values followed by capital letters (A–C) denote significant differences (*p* < 0.05) as a result of RAE concentration.

**Table 3:** The determination of free fatty acid (FFA; g·kg^−1^ lipids) content in fish muscle heated in the presence of red alga extract (RAE) *.

RAE Concentration	Heating Time (Days)				
	0	1	4	7	11
CTR	0.01 a (0.00)	0.01 aA (0.00)	0.02 aA (0.01)	0.11 bA (0.05)	0.96 cA (0.22)
RAE -1	0.01 a (0.00)	0.01 aA (0.00)	0.01 aA (0.00)	1.22 bB (0.26)	2.19 cB (0.25)
RAE -2	0.01 a (0.00)	0.01 aA (0.00)	0.02 aA (0.00)	2.36 bC (0.39)	4.30 cC (0.95)
RAE -3	0.01 a (0.00)	0.01 aA (0.00)	1.11 bB (0.19)	3.72 cD (0.45)	5.56 dC (1.16)

* Abbreviations of RAE concentrations as expressed in Table 1. The mean values of three independent determinations (*n* = 3); standard deviations are shown in brackets. Mean values followed by lowercase letters (a–d) denote significant differences (*p* < 0.05) as a result of heating time; mean values followed by capital letters (A–D) denote significant differences (*p* < 0.05) as a result of RAE concentration.

## Data Availability

All data are comprised within the article.
